# Transmission Dynamics and Genomic Epidemiology of Emerging Variants of SARS-CoV-2 in Bangladesh

**DOI:** 10.3390/tropicalmed7080197

**Published:** 2022-08-20

**Authors:** Md. Abu Sayeed, Jinnat Ferdous, Otun Saha, Shariful Islam, Shusmita Dutta Choudhury, Josefina Abedin, Mohammad Mahmudul Hassan, Ariful Islam

**Affiliations:** 1 EcoHealth Alliance New York, New York, NY 10018, USA; 2 Institute of Epidemiology, Disease Control and Research (IEDCR), Dhaka 1212, Bangladesh; 3 Department of Microbiology, Noakhali Science and Technology University, Noakhali 3814, Bangladesh; 4 Faculty of Veterinary Medicine, Chattogram Veterinary and Animal Sciences University, Chattogram 4225, Bangladesh; 5 Queensland Alliance for One Health Sciences, School of Veterinary Science, The University of Queensland, Gatton, QLD 4343, Australia; 6 Centre for Integrative Ecology, School of Life and Environmental Science, Deakin University, Melbourne, VIC 3216, Australia

**Keywords:** reproduction rate, phylogenetic analysis, clade GK, D614G, P681R

## Abstract

With the progression of the global SARS-CoV-2 pandemic, the new variants have become more infectious and continue spreading at a higher rate than pre-existing ones. Thus, we conducted a study to explore the epidemiology of emerging variants of SARS-CoV-2 that circulated in Bangladesh from December 2020 to September 2021, representing the 2nd and 3rd waves. We collected new cases and deaths per million daily data with the reproduction rate. We retrieved 928 SARS-CoV-2 sequences from GISAID and performed phylogenetic tree construction and mutation analysis. Case counts were lower initially at the end of 2020, during January–February and April–May 2021, whereas the death toll reached the highest value of 1.587 per million on the first week of August and then started to decline. All the variants (α, β, δ, η) were prevalent in the capital city, Dhaka, with dispersion to large cities, such as Sylhet and Chattogram. The B.1.1.25 lineage was prevalent during December 2020, but the B.1.617.2/δ variant was later followed by the B.1.351/β variant. The phylogeny revealed that the various strains found in Bangladesh could be from numerous countries. The intra-cluster and inter-cluster communication began in Bangladesh soon after the virus arrived. The prominent amino acid substitution was D614G from December 2020 to July 2021 (93.5 to 100%). From February–April, one of the VOC’s important mutations, N501Y substitution, was also estimated at 51.8%, 76.1%, and 65.1% for the α, β and γ variants, respectively. The γ variant’s unique mutation K417T was detected only at 1.8% in February. Another frequent mutation was P681R, a salient feature of the δ variant, detected in June (88.2%) and July (100%). Furthermore, only one γ variant was detected during the entire second and third wave, whereas no η variant was observed in this period. This rapid growth in the number of variants identified across Bangladesh shows virus adaptation and a lack of strict quarantine, prompting periodic genomic surveillance to foresee the spread of new variants, if any, and to take preventive measures as soon as possible.

## 1. Introduction

The novel viral pneumonia cases, characterized by high fever, dry cough, and occasionally catastrophic hypoxia, reported for the first time in Wuhan, China, in December 2019 had an epidemiological link with the live animal market [1]. The etiological agent was named severe acute respiratory syndrome coronavirus 2 (SARS-CoV-2) by the International Committee on Taxonomy of Viruses (ICTV) [2]. The first sequence of SARS-CoV-2 grouped the virus under the Sarbecovirus subgenus of the Coronaviridae family, typically categorized as Beta coronavirus [3,4]. By May 2021, the virus spanned over 203 countries of the world, with around 170 million cases and 0.35 million deaths [5], with a case fatality rate of 10% [6], which is higher than the seasonal flu outbreak (0.1–0.2%) [7]. SARS-CoV-2 is a highly recombinogenic virus, with 29903 nucleotides. The single-strand RNA virus has six functional open reading frames (ORFs), including replicas (ORF1a/ORF1b), spike (S), envelope, membrane, and nucleocapsids organized from 5′ to 3′ directions [8].

Mutations of the virus arise in almost every cycle. Frequent mutations, which are estimated roughly at 1.17–1.36 × 10^−3^ base substitutions per site per year [9] of the genomic composition, make the virus prone to continuous evolution, ultimately leading to the formation of new variants (variant of concern (VOC): Alpha/α (lineage B.1.1.7), Beta/β (lineage B.1.351), Gamma/γ (lineage P.1), Delta/δ (lineage B.1.617.2); variant of interest (VOI): Lambda (lineage C.37), Mu (lineage B.1.621), Epsilon (lineages B.1.429, B.1.427, CAL.20C), Zeta (lineage P.2), Theta (lineage P.3), Eta (lineage B.1.525), Iota (lineage B.1.526), Kappa (lineage B.1.617.1)). Several variants of SARS-CoV-2 have raised public health awareness due to their infectious nature. Among them, the α variant under the Pango lineage B.1.1.7 and GISAID clade GRY was first documented in the United Kingdom in September 2020 (a.k.a. 20B/501Y.V1 variant of concern (VOC) 202012/01). The β variant under the Pango lineage B.1.351 and GISAID clade GH/501Y.V2 was first detected in South Africa in May 2020. On the other hand, the VOC under P.1 lineage and GR clade/501Y.V3 was designated as Gamma γ VOC, and was isolated in Brazil. Another important VOC is δ, which originated in India in October 2020 and belongs to the B.1.617.2 lineage and G/478K.V1 clade (https://www.who.int/en/activities/tracking-SARS-CoV-2-variants/, accessed on 25 January 2022) [10]. The current circulating virus differs from the Wuhan variant at around 20 points in their genomes [11]. The different variants, such as the Brazilian variant P.1 lineage, emerged from the B.1.1.28 lineage due to 10 unique mutations in the virus genome, including spike protein (D614G), receptor-binding domain (RBD) (K417T, E484K, and N501Y), N-terminal domain (NTD) (L18F, T20N, P26S, D138Y, and R190S) and furin cleavage site (H655Y) [12]. The lineage B.1.1.7 emerged due to the major change in the amino acid asparagine by tyrosine at the 501 position of the spike protein, along with other mutations, increasing the transmissibility of the virus. Many more variants of the mother SARS-CoV-2 virus strain have also been reported in different countries and regions such as California, Nigeria, and India [13,14].

Phylogenetic analysis of the GISAID sequences identified different clusters named clades, where O was the ancestral type detected from Wuhan [15,16]. In early January and February 2020, the viruses were classified into clades 19A and 19B (L and S) [17]. Around 70% prevalent clade was L-type detected in the early stages from Wuhan, where S-type was also the ancestral type whose frequency decreased over the next few months. Again, A2a or Clade G, the ancestor of clades 20A-C, were identified in February, characterized by a specific non-synonymous mutation (D614G) in the spike protein [11]. Bangladesh experienced the start of the pandemic in March 2020 [18]. Since then, the devastating spread at the community level has continued. In the earlier period, the virus spread following a typical exponential growth curve with a higher reproduction rate [19]. Initially, to curb the transmission, the Government of Bangladesh implemented a countrywide lockdown procedure, but some unusual activities maintained the upward trend of the curve. However, by the end of July 2020, the curve sloped down, and the reproduction rate started to decline [20]. Meanwhile, the world started facing the challenges of different new variants of SARS-CoV-2. By December 2020, the new UK and South African variants were detected in Bangladesh [21]. In addition, the neighboring country, India, faced the havoc of COVID-19. After the end of May 2021, around 28 million people were affected, with the death of around 0.32 million [22]. The sequencing of the virus isolated from affected patients in India revealed a shared set of four genetic variant mutations in the genome of the core virus, which enhanced the effectiveness and transmissibility at an unbound rate [23].

In Bangladesh, the first α variant B.1.1.7 was detected at the end of January 2021 (GISAID). Since then, the case count has started to increase dramatically. Finally, the Government imposed the countrywide second lockdown procedure. Meanwhile, the sequencing of the virus identified 84 α-VOCs, which were first detected in the UK, 28 β-VOCs, which were first detected in South Africa, 44 δ-VOCs, which were first detected in India, 1 γ-VOC, which was first detected in Brazil/Japan, and 14 variant under investigation (VUI) Epsilon variants, first detected in Nigeria (www.GISAID.org, accessed on 15 January 2022). However, after two months of the lockdown, the Government decided against resuming normal activities. Therefore, in this study, we presented the case and death rates per million and showed the epidemiological and genomic diversity of SARS-CoV-2 variant strains in the Bangladesh population.

## 2. Materials and Methods

### 2.1. Temporal and Spatial Epidemiology of COVID-19

The Government of Bangladesh has been reporting daily COVID-19 cases through media briefings since 8 March 2020. The database is open for everyone to learn about the existing situation. We collected daily new cases and new deaths per million from 1 December 2020 to 15 September 2021. The number of cases and deaths per day was extracted from an online web portal (https://ourworldindata.org/coronavirus, accessed on 25 March 2022) because the data are open-sourced and the author declared that all visualizations, data and code produced by the web are completely open under the Creative Commons BY license and anyone has the permission to use, distribute and reproduce the data in any medium [24]. As the WHO and Worldometer coronavirus databases are also being updated on a real-time basis for every parameter, we included data from these two websites also [22,25]. The number of new cases and new death per day per million were presented graphically. From these data, we calculated the reproduction rate (R_t_) of COVID-19 for each day and presented them graphically [19]. We produced a geospatial map in ArcGIS 10.3, visualizing the spatial distribution of different VOCs according to the districts of Bangladesh [20].

### 2.2. Genomic Epidemiology

#### 2.2.1. Retrieval of Genomic Sequences and Metadata

From the Global Initiative on Sharing All Influenza Data (GISAID), we retrieved SARS-CoV-2 genomic metadata of Bangladeshi strains from 1 December 2020 to 15 September 2021. A total of 928 variant sequences were deposited during this time frame and were used in this study. We considered the genome length of 29,000 nucleotides for substitution mutation and phylogenetic analysis. This study did not include partial genomes with exceptionally high variation counts, gaps, or genomes that lacked a complete history of the patients/sampling location/collection date. Furthermore, genomic sequences that contained legionary characters (N, R, X, and Y) other than A, T, G, and C were omitted from the study [26]. We carried out the selection process by using manual sorting in MEGA 7. After that, we used pyfasta (https://github.com/brentp/pyfasta, accessed on 25 March 2022) to segment the whole genome into 2 different files, each with approximately 450 sequences. We aligned each file using the MAFFT web server, employing access modifiers, as described by Katoh et al. [27]. We used the complete genome sequence of the SARS-CoV-2 Wuhan-Hu-1 strain (accession NC 045512, version NC 045512.2). To filter all the ambiguous and low-quality sequences, we used the Sequence Cleaner (https://github.com/metageni/Sequence-Cleaner, accessed on 25 March 2022), applying specified parameters of minimum length (*m* = 3822), percentage N (*mn* = 0), maintaining all duplicates, and remove any ambiguous sequences. To find the gap-containing strains for deletion analysis, we used the SeqKit toolbox [28]. To delete the internal stop codon-containing sequences, we used the SEquence DAtaset builder [29]. Reference genomes from various nations were chosen based on the BLAST hit of the selected genomes (Supplementary File S1). The genomic sequences of the SARS-CoV-2 strains were aligned using the online Virus Pathogen Resource (https://www.viprbrc.org/, accessed on 25 March 2022) database and the MEGA 7 tool. The MSA file was then loaded with Jalview visualization software to remove redundancies from the sequences under study [30].

#### 2.2.2. Genome-Wide Analysis of Bangladeshi SARS-CoV-2 Variants

We initially analyzed the SARS-CoV-2 sequence by using “One Click Workflows” (https://ngphylogeny.fr/workflows/oneclick/, accessed on 4 October 2021), as described by Lemoine et al. [31]. We followed the standard process of phylogenetic tree building steps, including multiple alignments, alignment curation, tree formation, and visualization described by earlier researchers [32,33,34,35]. We used a web-based program, “Nextstrain” (https://clades.nextstrain.org, accessed on 25 March 2022), to generate the clade-designated tree. We used a subset of all the emerging virus sequences for making variant-specific phylogenetic trees using the neighbor-joining methods described by Islam et al. [19]. Furthermore, to identify the possible transmission routes of every reported variant in Bangladesh, all interactive phylogenetic reconstructions were carefully evaluated to discover the likely ancestral sources of the Bangladeshi SARS-CoV-2 variant genomes. Each branch tip bearing a Bangladeshi genome found in accessible Nextstrain clades was recognized in the original phylogeny. That branch was then examined backwards in the tree, until all the closely related exogenous strains and their countries of origin were discovered. We used the automated global map-based analysis in auspice version 0.5.0 (https://auspice.us, accessed on 25 March 2022) by Nextstrain [36] to visualize the transmission paths.

Finally, the aligned sequences were viewed for mutation analysis with MEGA 7 and the Virus Pathogen Resource (https://www.viprbrc.org/, accessed on 25 March 2022) to identify the deletions and insertions compared to the reference genome. Furthermore, mutations that result in amino acid substitutions were investigated using the Wuhan reference sequence and the GISAID platform, which included the CoVserver enabled by GISAID in the GIDAID EpiCoV database, as well as a blast (https://blast.ncbi.nlm.nih.gov/, accessed on 25 March 2022) of the entire genome and individual proteins [37].

## 3. Results

### 3.1. Epidemiology of COVID-19 in Bangladesh

#### 3.1.1. Temporal Distribution of COVID-19 Cases and Deaths during 2nd and 3rd Wave

**Figure 1:**
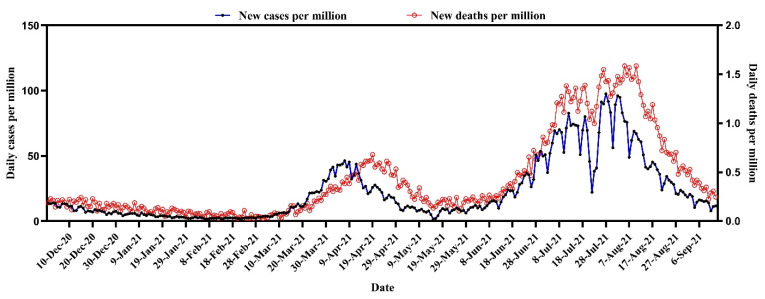
Temporal trend of COVID-19 cases and deaths in Bangladesh (per million).

At the end of 2020, COVID-19 cases were 6.157 per million, and the death rate was 0.17 per million in Bangladesh. During March–April and June–July 2021, we witnessed the 2nd and 3rd waves of COVID-19, respectively. The cases started increasing on 2 March, and reached the highest on 7 April 2021. Then, the case counts declined gradually and reached the lowest count in the middle of May. After that, the case count suddenly increased from the middle of June and reached 53.5 cases per million at the end of the month. From 1 July, the cases per million increased and peaked at 97.5 on 28 July. From August onwards, the new cases reduced slowly and reached 11.7 cases per million on 15 September 2021 (Figure 1).

Similarly, the number of deaths increased from the end of March to the end of April. The death count reached the optimum level of around 0.680 death per million cases on 19 April. Then, there was a gradual declining trend with a zigzag pattern. However, after 13 June, the number of deaths increased again and reached 0.698 per million on 30 June. From the start of July, the deaths per million followed an upward trend to reach its highest at 1.551 on 27 July. Again, the death toll decreased to 1.275 per million on 30 July, then started floating and reached its highest at 1.587 per million on 10 August, then finally declined and reduced to 0.247 on 15 September 2021 (Figure 1).

#### 3.1.2. Daily Reproduction Rate of COVID-19 Cases during 2nd and 3rd Wave

**Figure 2:**
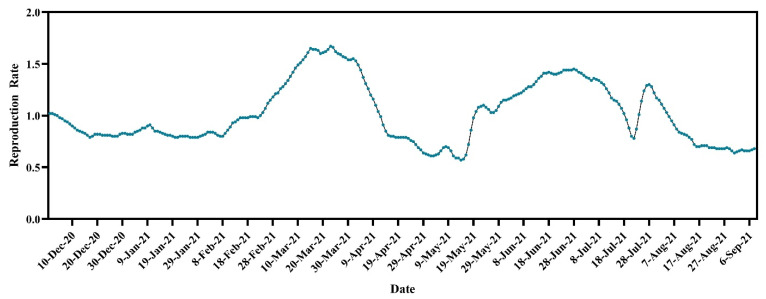
Temporal dynamics of reproduction rate of SARS-CoV-2 cases in Bangladesh (https://ourworldindata.org/coronavirus, accessed on 15 September 2021).

The overall R_t_ was just over one (1.02) on 1 December 2020. The R_t_ decreased to less than one and maintained a plateau until 22 February 2021. After that, the reproduction rate increased by over one and reached its highest at 1.67 on 23 March 2021. Then, the reproduction rate started declining and dropped below one again and reached its lowest value of 0.57 on 14 May 2021. However, from May 20, the reproduction rate started to increase and reached 1.45 on 28 June 2021. In the first half of July, the R_t_ was over one and decreased below one on 19 July. The R_t_ was less than one until 23 July and then again increased above one during the last few days of July, which persisted until 4 August. After that, the R_t_ decreased to less than one, and this trend persisted until 15 September 2021 (Figure 2).

#### 3.1.3. Spatial Distribution of Emerging Variants of COVID-19 in Bangladesh

**Figure 3:**
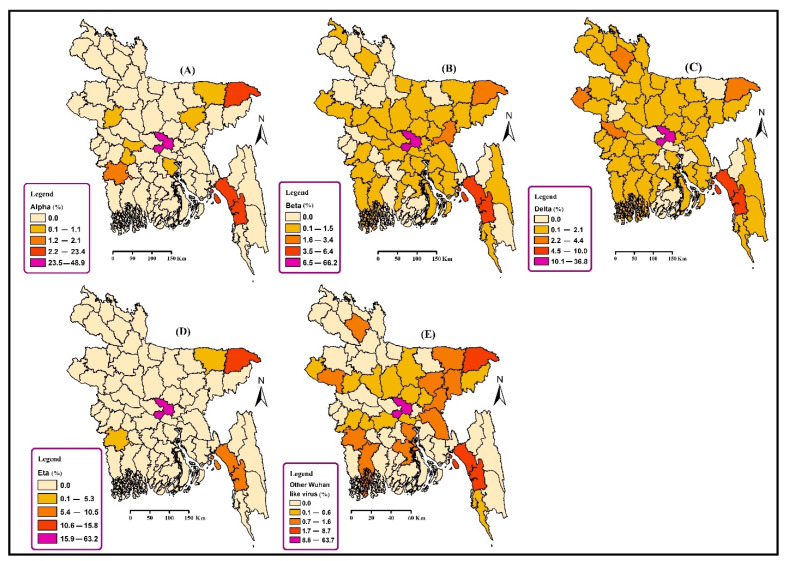
Spatial distribution of (**A**) Alpha variants (%); (**B**) Beta (%); (**C**) Delta (%); (**D**) Eta (%) variants of SARS-CoV-2; and (**E**) other Wuhan-like strains (%) in Bangladesh from December 2020 to 15 September 2021. We have omitted the Gamma variant as only one sequence has been reported in GISAID and visualization of a single virus might mislead the geographic distribution of the variant.

The highest percentage of all the variants, α, β, γ, and δ, was found in the Dhaka district. In addition, the α variant was also prevalent in the Sylhet and Chattogram districts. The Chattogram district also had a higher frequency of the β variant. Another peripheral district, Nawabganj, had a higher number of cases due to the δ variant (Figure 3). The frequency of the area-specific VOCs is given in Supplementary File S2.

#### 3.1.4. Clade and Lineage Diversity of SARS-CoV-2 in Bangladesh

**Figure 4:**
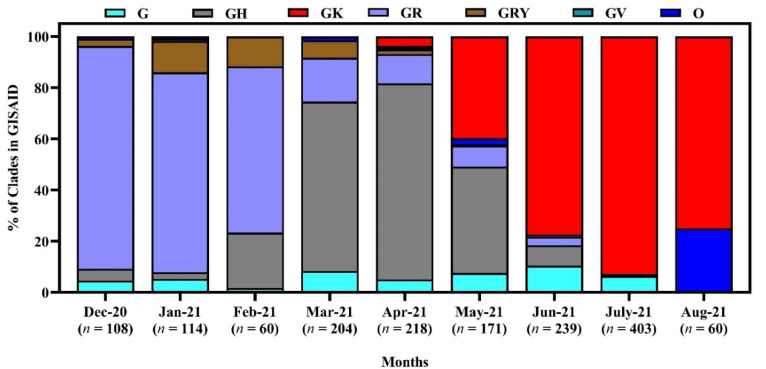
Clade diversity of SARS-CoV-2 in Bangladesh from December 2020 to September 15, 2021. Here, the clades are based on the marker variants **G**: C241T,C3037T,A23403G includes S-D614G; GH: C241T,C3037T,A23403G,G25563T includes S-D614G + NS3-Q57H; GK: C241T,C3037T,A23403G,C22995A S-D614G + S-T478K; GR: C241T,C3037T,A23403G,G28882A includes S-D614G + N-G204R; GRY: C241T,C3037T,21765-21770del,21991-21993del,A23063T,A23403G,G28882A includes S-H69del, S-V70del, S-Y144del, S-N501Y + S-D614G + N-G204R; GV: C241T,C3037T,A23403G,C22227T includes S-D614G + S-A222V; O: Includes S: C8782T,T28144C includes NS8-L84S + L: C241,C3037,A23403,C8782,G11083,G26144,T28144 + V: G11083T,G26144T NSP6-L37F + NS3-G251V [38].

We have studied 928 genome sequences of SARS-CoV-2 reported from Bangladesh from December 2020 to 15 September 2021. From December 2020 to February 2021, the GR clade was the most prevalent (87.04, 77.7, 65%, respectively) among all the clades. However, in March and April, GH was Bangladesh’s most highly distributed clade. The scenario changed in May and June, when most cases were affected by strains under the clade GK. The GK clade prevailed in Bangladesh until July 2021 (Figure 4).

**Figure 5:**
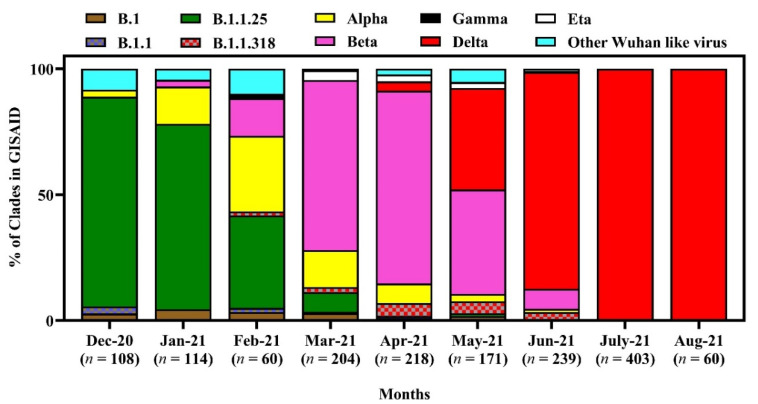
Lineage diversity of SARS-CoV-2 in Bangladesh from December 2020 to 15 September 2021.

A variety of lineages have reigned in Bangladesh since December 2020. In December 2020, January, and February 2021, the most prevalent lineage was B.1.1.25 (83.3, 74.1, and 38.3%, respectively). However, along with B.1.1.25, another lineage, the α variant B.1.1.7, also increased in Bangladesh during February 2021. However, in March and April, the β variant B.1.351.3 was the most dominant (67.8% and 76.7%, respectively), whereas lineage B.1.617.2 (δ variant) was predominant in May, June and July 2021 (43.2, 87.6 and 100%, respectively) (Figure 5).

### 3.2. Transmission Route of Emerging Variants in Bangladesh

**Figure 6:**
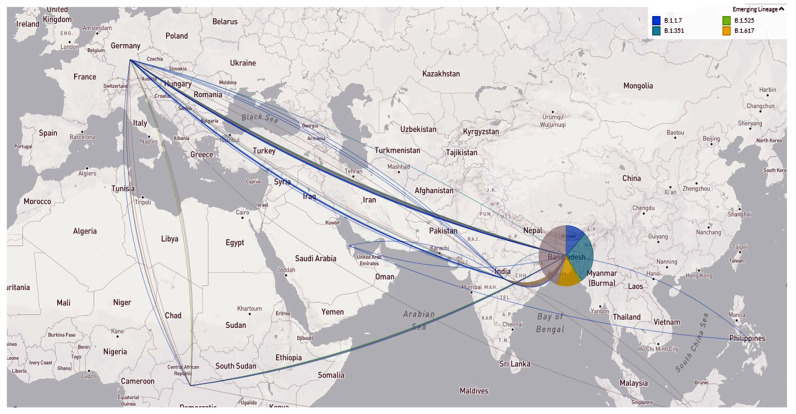
Emerging variant’s geographic transmission lines to Bangladesh (live display at nextstrain.org/ncov, accessed on 15 September 2021).

Figure 6 shows the transmission pathways of the emerging variants from different countries to Bangladesh. The VOC B.1.1.7 was mainly introduced into Bangladesh from European countries, such as Germany, UAE, African countries, and the Philippines. The B.1.525 entered mainly from African countries to Bangladesh. In addition, the Delta (B.1.617.2) variant entered from India (Figure 6).

### 3.3. Genomic Epidemiology of SARS-CoV-2 Variant of Concern (VOC) in Bangladesh

#### 3.3.1. 20I/501Y.V1, Alpha Variants/B.1.1.7 in Bangladesh

**Figure 7:**
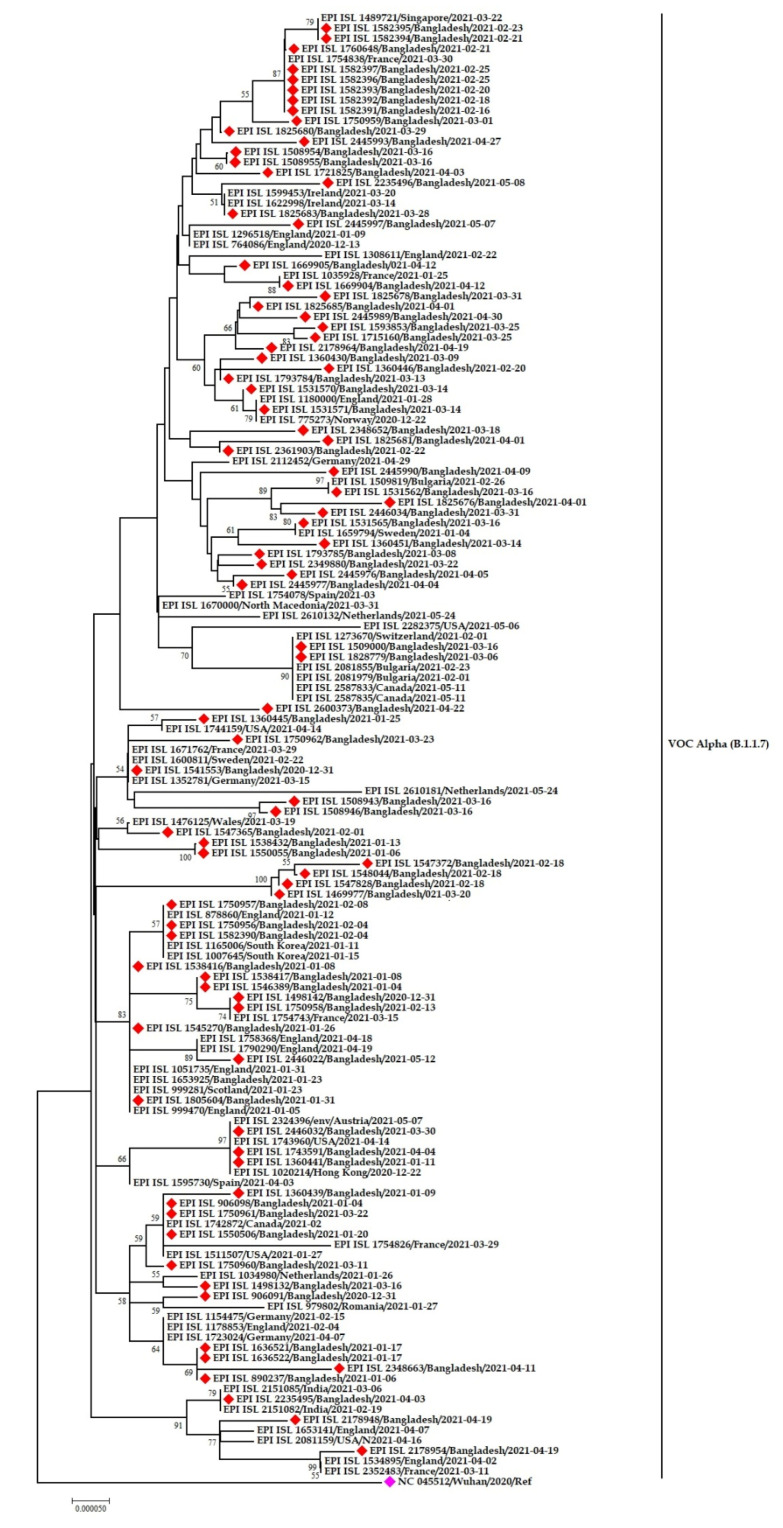
Phylogenetic analysis of emerging Alpha variants of SARS-CoV-2 in Bangladesh. Here, red diamond dots denote Bangladeshi Alpha variants SARS-CoV-2 viruses, and pink dots denote the Wuhan-Hu-1 viruses.

The Alpha variants/B.1.1.7 of SARS-CoV-2 in Bangladesh showed fourteen different instances of clustering with sequences from around the world. Most of the Bangladeshi strains clustered with virus sequences from Singapore, France, Ireland, England, Norway, Bulgaria, Switzerland, South Korea, Netherlands, Austria, Hong Kong, India, and Romania. Some of the reported sequences clustered with sequences from the USA, Canada, Germany, and Sweden (Figure 7).

Figure 7 also suggests multiple viral introductions into Bangladesh from several countries. The sample EPI-ISL-1750957 from Bangladesh is closely related to the sample EPI-ISL-878860 isolated in England. Another Bangladeshi sample EPI-ISL-1750958 is highly similar to the sample EPI-ISL-1754743 from France. The two samples EPI-ISL-1750960 and EPI-ISL-1550506 are close to the EPI-ISL-1511507 sample from the USA, EPI-ISL-1742872 from Canada, and EPI-ISL-1743960 from the USA. The distinct Bangladeshi sample EPI-ISL-1669904 has also shown similarities with samples from France (EPI-ISL-1035928).

#### 3.3.2. 20H/501Y.V2, Beta Variants in Bangladesh

**Figure 8:**
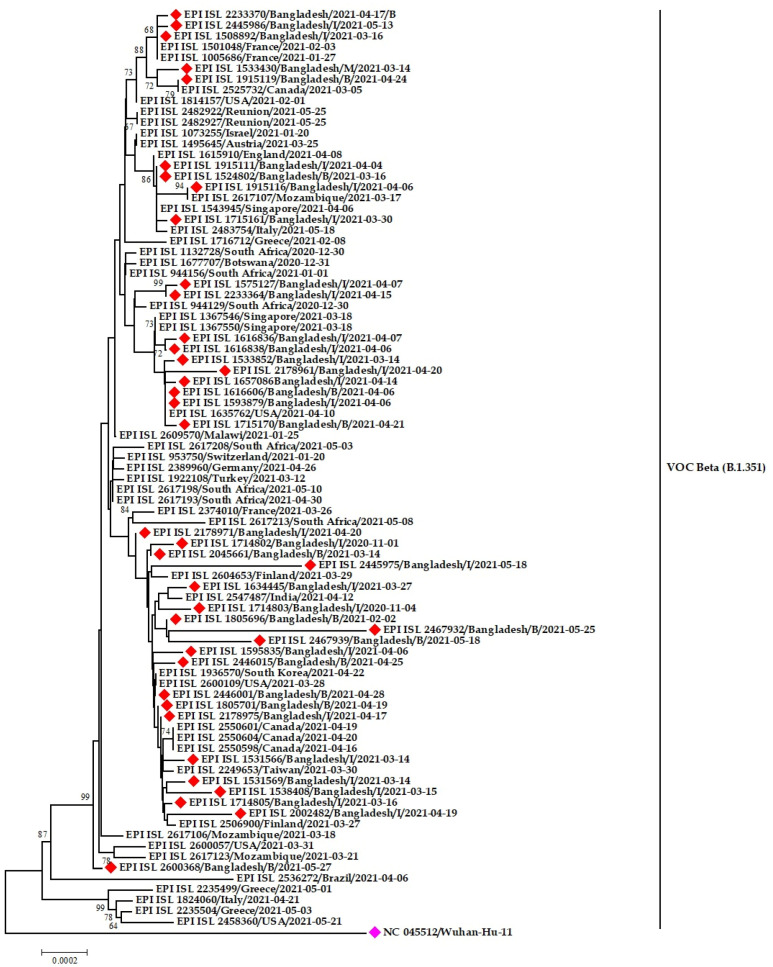
Phylogenetic analysis of emerging Beta variants of SARS-CoV-2 in Bangladesh. Here, red diamond dots denote Beta variant SARS-CoV-2 viruses, and pink dots denote Wuhan-Hu-1 viruses.

**Figure 9:**
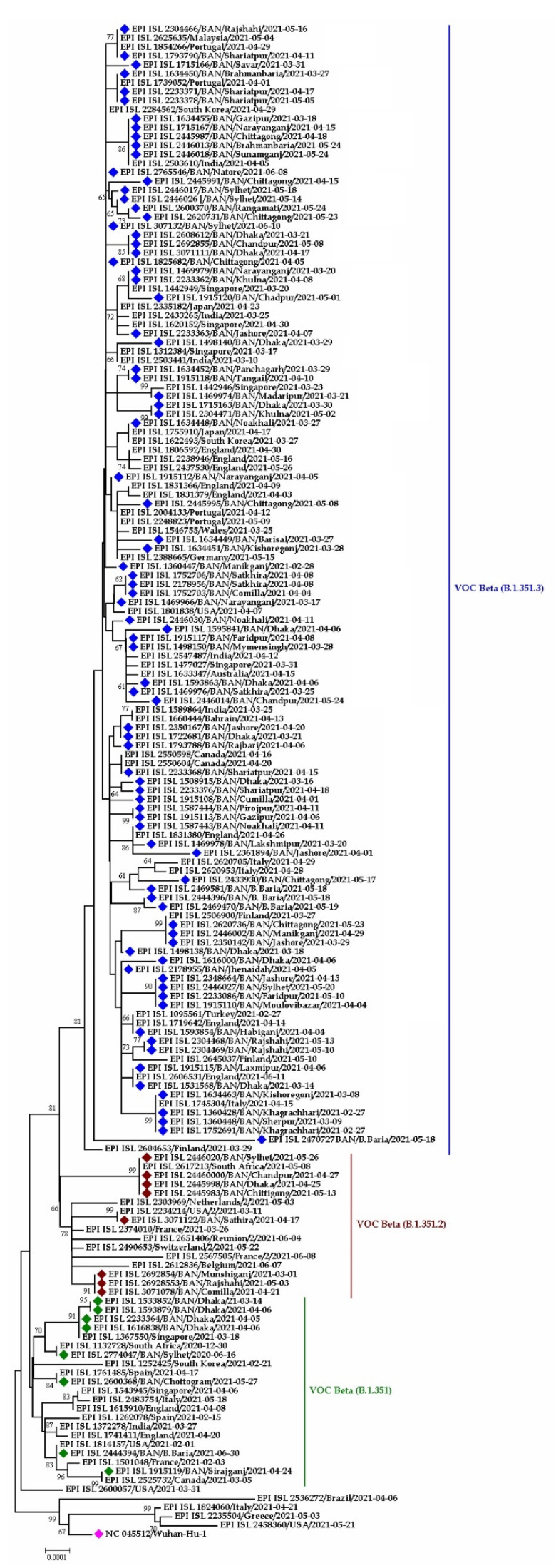
Phylogenetic analysis of sub-lineage Beta variant B.1.351.3 in Bangladesh. Here, blue dots denote Beta (B.1.351.1) SARS-CoV-2 viruses, and maroon, green and pink dotes denote B.1.351.2, B.1.351, and Wuhan-Hu-1 viruses, respectively.

All the three-pangolin lineages of Beta variants (B.1.351, B.1.351.2 and B.1.351.3) were observed in Bangladesh (Figure 8 and Figure 9). The B.1.351 circulated in Dhaka, Chattogram, and Sirajgonj from March to May 2021. Later in June, it was found in the Brahmanbaria and Sylhet districts. The source of introduction of B.1.351 was likely South Africa, Canada, Spain, USA.

The Bangladeshi strains clustered with virus sequences were reported from mainly European countries and the Middle east. The Bangladeshi Beta variants have a likeness to virus sequences reported from England, Germany, Switzerland, Scotland, Italy, Turkey, and Jordan (Figure 8).

B.1.351.2 was found in Munshigonj in March but in Dhaka, Chandpur, Sathira and Comilla in April 2021. Later in May, it was distributed to other districts, such as Rajshahi, Chattogram and Sylhet. On the other hand, the sub-lineage B.1.351.3 of Beta variants formed twelve different clusters. This lineage was circulating in Manikgonj and Khagrachhari during February 2021. The strain from Manikgonj stands alone in the phylogenetic tree without resembling other strains. However, the strain from Khagrachari later spread to Sherpur and Kishoregonj districts in March. The strain spread from Dhaka to other districts during March, April, and May (Figure 9).

#### 3.3.3. VOC G/452R.V3 Delta Variant (B.1.617.2) in Bangladesh

**Figure 10:**
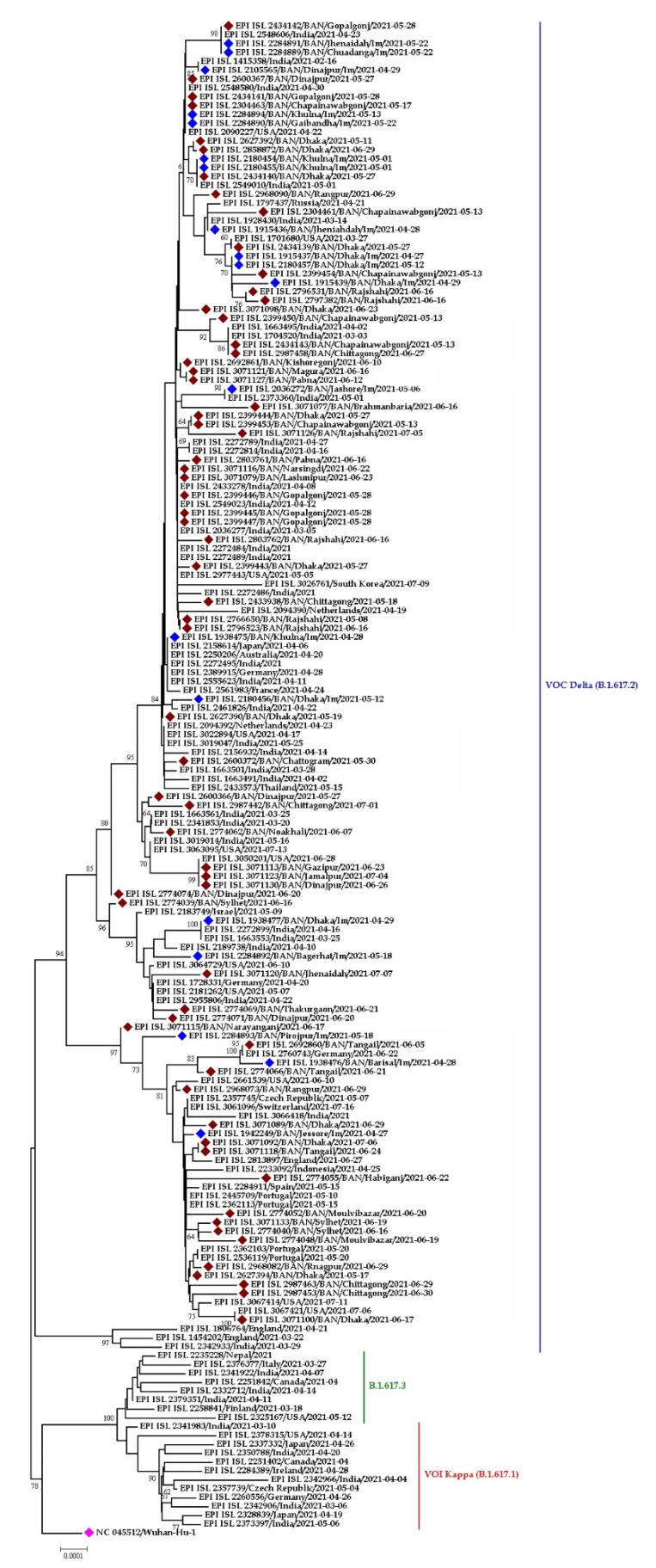
Phylogenetic analysis of sub-lineage of Delta variant (B.1.617.2) in Bangladesh. Here, maroon dots denote community transmission of Delta variant SARS-CoV-2 viruses; blue and pink dotes denote imported SARS-CoV-2 Delta variant and Wuhan-Hu-1 viruses, respectively. B.1.617.3 and B.1.617.1 were used as out group here.

Among the three three pangolin lineages of the Delta variant, only one (B.1.617.2) was detected in Bangladesh. This variant was thought to be introduced in April in Dinajpur, Jhenaida, Dhaka, or Khulna. The strains from Dhaka showed nucleotide similarity with a strain (EEPI ISL 2189738) from India. The phylogeny illustrates the direct relationship between strains from India and Dinajpur, Chapai-nawabgonj, Khulna, Dhaka, and Chattogram. After arriving in Bangladesh, the virus began to spread at a neighborhood level and expanded throughout the county quickly (Figure 10). In the phylogenetic tree (Delta V), it was discovered that when one virus was detected in Jashore, it was also reported in other parts of the country, including Noakhali, Laxmipur, Gopalganj, and Narshingdi, demonstrating community-level transmission of Indian-originated viruses. Another cluster supports community transmission of the Delta variant strain of SARS-CoV-2 virus from Jashore to other districts (Dhaka, Chattogram, Sylhet, Habigonj, Tangail, and Rangpur) (Figure 10).

#### 3.3.4. VUI G/484K.V3 and VOC GR/501Y.V3 Variants in Bangladesh

**Figure 11:**
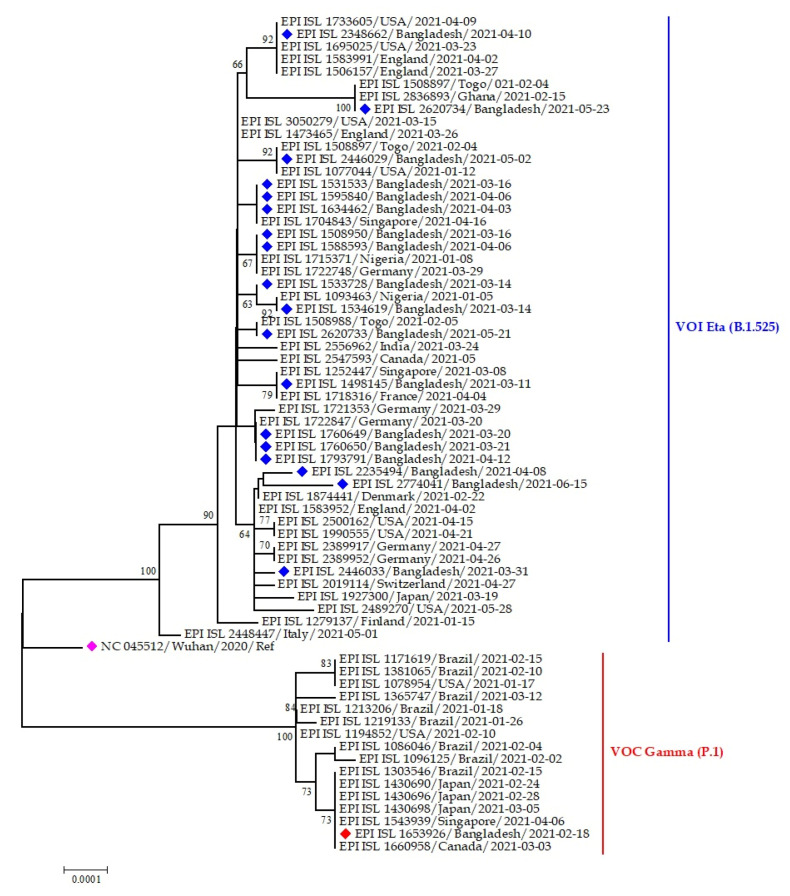
Phylogenetic analysis of emerging Eta and Gamma variants of SARS-CoV-2 Bangladeshi isolates. Here, blue dots denote Eta variant SARS-CoV-2 viruses; red and pink dotes denote Gamma variant and Wuhan-Hu-1 viruses, respectively.

The Eta variants of SARS-CoV-2 Bangladeshi strains clustered with virus sequences were reported from the USA, England, Togo, Ghana, Singapore, Nigeria, Germany, and France, whereas only one Gamma variant was reported from Bangladesh, which clustered with strains from Brazil, Canada, Japan, and Singapore (Figure 11).

#### 3.3.5. Point Mutation Analysis of SARS-CoV-2 in Different Time Period in Bangladesh

The highest amino acid substitution was D614G from December 2020 to July 2021. This mutation was present in almost all the sequences from Bangladesh (ranging from 93.5 to 100%). The overall P681H mutation was detected at a low percentage (ranging from 4.8 to 35.7%) from Dec to July. E484K was found at the highest proportion during March (64.7%) and April (65.1%), 2021. At the same time, K417N was more prevalent in March (65.2%). N501Y is one of the critical mutations for the α, β and γ variants. During February, March, and April, the N501Y substitution was found at 51.8, 76.1, and 65.1%, respectively.

**Figure 12:**
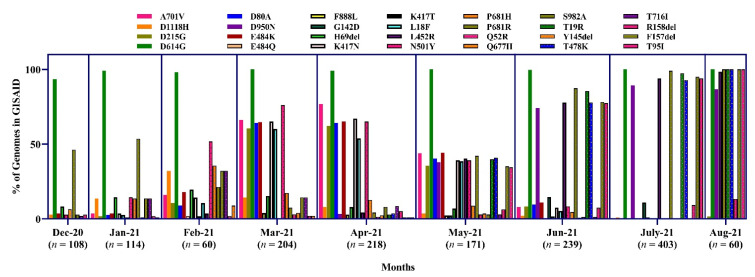
Proportion of amino acid mutations in the spike protein of SARS-CoV-2 sequences in Bangladesh.

The K417T mutation was detected only at 1.8% in February. On the other hand, another frequent mutation was P681R in June (88.2%) and July (100%). Some other significant mutations, D950N and L452R. D950N, L452R, T478K, were frequently increased from May and detected in 82.4, 94.1, and 88.2% of sequences, respectively, in July (Figure 12).

#### 3.3.6. Variant Specific Mutation Analysis of SARS-CoV-2 in Bangladesh

**Figure 13:**
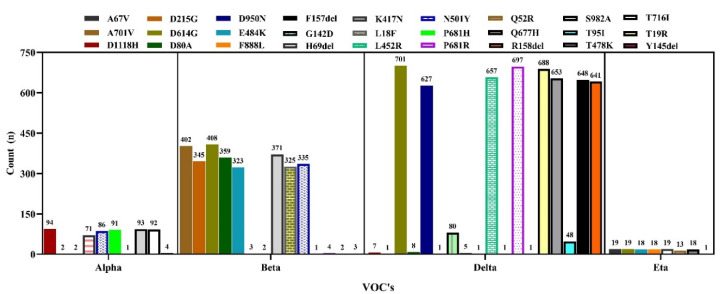
Frequency of substitution mutations in the spike protein of VOCs’ sequences in Bangladesh.

Again, we present the variant-specific substitution mutations in the spike protein in Figure 13. In the case of the Alpha variant (*N* = 94), the most frequent substitution mutation was observed at D1118H (*n* = 94), followed by S982A (*n* = 93) and P681H (*n* = 91). In the case of the Beta variant, the substitution mutation D614G (*n* = 408) was more commonly observed, followed by A701V (402) and K417N (371). In the Delta variant (*N* = 702), the highest substitution was D614G (*n* = 701), followed by some other unique mutation, including T19R (*n* = 688), R158del (*n* = 641) and D950N (*n* = 627). In the Eta variant (*N* = 19), the most frequent substitution was A67V (*n* = 19) (Figure 13). We detected only one Gamma variant, which has been omitted from this analysis.

## 4. Discussion

### 4.1. Spatial and Temporal Epidemiology of COVID-19 in Bangladesh

In 2020, Bangladesh was able to combat the initial SARS-CoV-2 outbreak wave successfully [39]. We observed that the second wave of COVID-19 cases gradually started to spreadin early March, with a notable spike in cases occurring around the middle of April. From February to March 2021, Bangladesh began to experience the severity of the second wave of COVID-19 cases and deaths related to the β variant (also known as the South African variant) [40]. A nationwide lockdown successfully reduced SARS-CoV-2 transmission during this time (12 to 15 April 2021), from daily 3.15 to 2.35 cases per 100,000 population [41]. Although the Government started mass vaccination on 7 February 2021, ninety percent of all variants during April and May 2021 were Beta variants [42]. However, the double mutant Delta variant (B.1.617.2) was discovered for the first time in Bangladesh on May 8th, 2021; since then, the third wave of COVID-19 appeared with a sharp increase at the end of June 2021, with 68% prevalence [43]. Additionally, 20–55% of those who had previously recovered from COVID-19 caused by the other variant were infected by this strain [44]. The variant also exerted roughly eight-fold less sensitivity to Oxford-AstraZeneca and Pfizer-BioNTech vaccine-generated immunity compared to the Alpha variant, according to a number of epidemiological and in vitro findings [45]. Therefore, the protective effects of vaccination were not observed at that time; however, the mass vaccination program has proven to be successful, with the reduction in daily new cases and deaths in recent times. In our analysis, we found a peak at the third wave by the end of July when only 0.94% people of this country received their first shot of the vaccine, which was much lower that other countries across the world [24]. Moreover, the reproduction rate increased simultaneously, along with the progression of the 2nd and 3rd waves. The timeline of the 2nd and 3rd waves of COVID-19 in Bangladesh corresponds with the introduction of several variants of concern into the country [40].

Several studies reported that participants have more knowledge about the disease’s risk; thus, they wear masks in public places [46,47]. However, Bangladesh still faced the second and third waves of COVID-19. Considering the vast population of Bangladesh, only a small number of test centers are available. Furthermore, a longer time is needed for the COVID-19 test results to return [48]. Almost 19% of patients were asymptomatic when they tested positive for COVID-19 during the second wave. In addition, a similar percentage of patients did not know how they became infected, and both urban and rural areas were affected equally [49]. So, people roam freely before testing positive for COVID-19 and spread the infection to other exposed humans. Additionally, if the residents practiced strict preventive measures, there would not be a surge of COVID-19 cases in Bangladesh. The 2nd and 3rd waves resulted from people not maintaining social distance, unwillingness to wear masks, and scarcity of vaccines for susceptible populations. All these factors collectively favor the rapid spread of the virus and the emergence of a higher number of cases in Bangladesh.

With the progression of the global SARS-CoV-2 pandemic, the new variants are becoming more infectious and continue spreading at a higher rate in contrast to pre-existing variants, due to changes in the virus genomic composition [50,51]. All the variants, α, β, γ, δ, and η, were most prevalent in the central districts, such as Dhaka. All the VOCs were more prevalent in Dhaka because it is the port of entry for millions of people every day. Dhaka is the capital city of Bangladesh. It connects other districts via rail, air, and waterways. Dhaka has an international airport, a few ferry ghats, and several rail stations. These ports of entry make Dhaka vulnerable to all the emerging variants. So, it is not surprising to observe the presence of all the variants in the capital district. The only exception was the δ variant, which was distributed to the peripheral districts along with Dhaka. This variant was responsible for Bangladesh’s 3rd wave of COVID-19 [52]. The δ variant originated in the neighboring country India [53]. The peripheral districts, such as Comilla, Dinajpur, and Sylhet, have borders with India. People come and go through the border daily for various purposes. As a result, the Delta variant was dominant in those peripheral districts. In addition, dustbins are unavailable in rural areas, so people dump their wastes on roadside pits or drain water bodies, and vacant plots near houses [54]. These factors also contribute to viral spreading in peripheral districts.

There was a changing pattern in the clade prevalence in Bangladesh from December 2020 to September 2021. Although initially, the clade GR was predominant, later it reduced, and there was a rise in clade GK in Bangladesh. A similar changing pattern of lineages from B.1.1.25 to B.1.617.2 has been observed over the same period. The changing pattern in the lineage’s distribution corresponds to the gradual spread of β and δ variants during the 2nd and 3rd waves of COVID-19 in Bangladesh [55].

### 4.2. Transmission Dynamics and Phylogeny of Emerging Variants in Bangladesh

The investigative genomic analysis of the emerging variants confers the high prevalence of α, β, and δ, the significant determining variants for Bangladesh’s second and third wave [21]. We revealed the possible origin and transmission route of the emerging variants in Bangladesh through Nexstrain and phylogenetic analyses. The emerging variants’ introduction to Bangladesh was through European and African countries. This fact was again re-confirmed based on the evidence presented in the phylogenetic trees. The variants circulating in Bangladesh are of European origin, mainly England, France, Germany, Ireland, Italy, and the USA. However, some viral sequences demonstrate similarities with other Asian countries, including Singapore, Japan, and South Korea, which has already been established by earlier studies [56,57]. All the clustering of the Bangladeshi sequences indicates the virus’s transmission from expatriates to the community, which might be due to an improperly structured quarantine facility for travelers in Bangladesh. Multiple introductions have been recorded from Italy, India, and the UK [58]. The clustering of α and β variants of SARS-CoV-2 in Bangladesh illustrated that the virus reached the community transmission level, and only one Eta variant indicates the lack of community transmission of the emerging Eta variants.

We also conducted mutation analyses for all the Bangladeshi sequenced strains of SARS-CoV-2 deposited in the GISAID. D614G is the most dominant mutation in the Bangladeshi strains [19]. Similarly, the mutations that are important for several VOCs were recorded from the sequences of infected patients and environments in Bangladesh [19,59,60]. The Alpha, Beta and Gamma variants were common during March and April, but later in June–July, the Delta variant replaced other VOCs. Therefore, Delta variant-related mutations were increasing gradually [55,60].

The global SARS-CoV-2 vaccines are currently available for human immunization. Moreover, variant-proof COVID-19 vaccines and pan-Beta coronavirus vaccines are in the development stage that could, in the future, protect against multiple COVID-19 variants and other Beta coronaviruses, such as MERS and SARS [61,62]. Although there are around 115 vaccines that have been reported, among which some are available for immunization, about 53.1% of people have received at least one dose of any COVID-19 WHO-approved vaccine across the globe, whereas in Bangladesh, around 87 million people only received their vaccine in November 2021 [63]. However, the overall immunization rate depends on the people’s acceptance of vaccines, which is still questionable [64]. In addition, a considerable level of people need to be vaccinated to attain herd immunity [65]. Therefore, the government, policy planners, and stakeholders should take action with regard to people’s apprehensiveness towards immunization against SARS-CoV-2 to break the transmission dynamics.

## 5. Conclusions

COVID-19 started to impact human lives in Bangladesh in the first quarter of 2020. From the end of 2020, the case counts were lower. Nevertheless, during March–April and June–July 2021, we witnessed the 2nd and 3rd waves of COVID-19. Although initially, Bangladesh dealt with only COVID-19, from December 2020, several variants of concerns started to arise. The variants were prevalent in Dhaka, the capital city, with frequent dispersion to other large cities, such as Sylhet and Chattogram. Simultaneously, the Delta variants were prevalent in border districts, as they originated in India. Initially, the GR clade was higher, and then gradually, clade GK replaced it. Similarly, lineage B.1.1.25 was prevalent during December 2020; however, later, the Delta variant prevailed in Bangladesh. Detection of specific mutations related to specific variants further confirms the results. There are also phylogenetic relations between Bangladeshi strains and strains from other countries. Thus, we recommend frequent genomic surveillance to forecast the spreading of new variants, if any, and to take preventive steps as soon as possible.

## Data Availability

All data generated or analyzed during this study are included in this published article (and its Supplementary Information files).

## Supplementary Materials

The following supporting information can be downloaded at: https://www.mdpi.com/article/10.3390/tropicalmed7080197/s1, File S1: SARS-CoV-2 genome sequence metadata; File S2: District wise frequency of VOC’s.
